# The Novel Multiple Inner Primers-Loop-Mediated Isothermal Amplification (MIP-LAMP) for Rapid Detection and Differentiation of *Listeria monocytogenes*

**DOI:** 10.3390/molecules201219787

**Published:** 2015-12-03

**Authors:** Yi Wang, Yan Wang, Aijing Ma, Dongxun Li, Lijuan Luo, Dongxin Liu, Shoukui Hu, Dong Jin, Kai Liu, Changyun Ye

**Affiliations:** 1 State Key Laboratory of Infectious Disease Prevention and Control, National Institute for Communicable Disease Control and Prevention, Collaborative Innovation Center for Diagnosis and Treatment of Infectious Diseases, Chinese Center for Disease Control and Prevention, Changping, Changbai Road 155, Beijing 102206, China; wildwolf0101@163.com (Y.W.); wangyan@icdc.cn (Y.W.); aiaijingjing@yeah.net (A.M.); gongweirenluo@163.com (L.L.); shoukuihu@163.com (S.H.); jindong@icdc.cn (D.J.); liukai@icdc.cn (K.L.); 2 Changping District Center for Disease Control and Prevention, Changping, Beijing 102200, China; xuner1989@163.com; 3 Pathogenic Biology Institute, University of South China, Hengyang 421000, Hunan, China; liucommon@126.com

**Keywords:** MIP-LAMP, LAMP, LoD, *L. monocytogenes* detection, *HlyA* gene amplification

## Abstract

Here, a novel model of loop-mediated isothermal amplification (LAMP), termed multiple inner primers-LAMP (MIP-LAMP), was devised and successfully applied to detect *Listeria monocytogenes*. A set of 10 specific MIP-LAMP primers, which recognized 14 different regions of target gene, was designed to target a sequence in the *hlyA* gene. The MIP-LAMP assay efficiently amplified the target element within 35 min at 63 °C and was evaluated for sensitivity and specificity. The templates were specially amplified in the presence of the genomic DNA from *L. monocytogenes*. The limit of detection (LoD) of MIP-LAMP assay was 62.5 fg/reaction using purified *L. monocytogenes* DNA. The LoD for DNA isolated from serial dilutions of *L. monocytogenes* cells in buffer and in milk corresponded to 2.4 CFU and 24 CFU, respectively. The amplified products were analyzed by real-time monitoring of changes in turbidity, and visualized by adding Loop Fluorescent Detection Reagent (FD), or as a ladder-like banding pattern on gel electrophoresis. A total of 48 pork samples were investigated for *L. monocytogenes* by the novel MIP-LAMP method, and the diagnostic accuracy was shown to be 100% when compared to the culture-biotechnical method. In conclusion, the MIP-LAMP methodology was demonstrated to be a reliable, sensitive and specific tool for rapid detection of *L. monocytogenes* strains.

## 1. Introduction

*Listeria monocytogenes* (*L. monocytogenes*) is a facultative intracellular pathogen that is widespread in the environment and is common in various food products, such as meat, fish, vegetables, milk, and dairy products [1,2]. The organism is characterized by its ability to multiply at low temperatures and survive in harsh conditions, such as high salt concentrations as well as a wide range of pH values [3]. *L. monocytogenes* is the etiological agent of listeriosis, with manifestations in both humans and animals ranging from flu-like early symptoms, septicaemia, encephalitis, meningitis, central nervous system damage, and abortion to stillbirth [4].

Human listeriosis, one of the most virulent and life-threatening food-borne illnesses, is associated with the consumption of *L. monocytogenes*—contaminated foods [5]. The most vulnerable groups include older adults, newborn infants, immunocompromised patients and pregnant women [6]. Although listeriosis is less common than other foodborne diseases, it has a high mortality rate approaching 30%, which far exceeds that of other foodborne pathogens [7,8]. Therefore, it is critical to develop a rapid, inexpensive, highly specific and sensitive method to screen *L. monocytogenes*.

Conventional methods for the isolation and identification of *L. monocytogenes* are culture-based and require a relatively long period of time for incubation procedures, several “hands-on” manipulations and biochemical confirmation to a species level [9]. Thus, some of the disadvantages of these methods are being time consuming, labor-intensive, and not always reliable. As an alternative diagnostic technique, several PCR-based assays have been developed for the detection of *L. monocytogenes*, including conventional PCR and real-time PCR [10,11,12,13]. However, established PCR-based techniques rely on sophisticated apparatus, such as temperature-regulating equipment, which restricts their widespread application in resource-poor settings [14,15,16]. Therefore, there is a growing demand for devising a novel strategy for rapid, robust and sensitive identification of *L. monocytogenes* using simple equipment.

Recent developments in isothermal amplification assays provide a variety of nucleic acid signal-amplification strategies, among which loop-mediated isothermal amplification (LAMP) is a promising candidate due to its rapidity, simplicity, high efficiency and specificity [17,18,19,20]. The methodology has been demonstrated to be useful for regulatory and epidemiological surveillance purposes, where the trace amounts of target templates could be difficult to detect [21]. In order to be more valuable in nucleic acids-based diagnostics and widely applied in various fields, the sensitivity of LAMP technology must be markedly enhanced. Thus, it is a challenging task to modify the LAMP technique to a new model with higher sensitivity, specificity and efficiency for regular diagnosis, food hygiene inspection, point-of-care testing and more.

In the present paper, we developed a novel mode of LAMP, termed multiple inner primers-LAMP (MIP-LAMP), in which the nucleic-acid sequences of sample DNA were detected with high sensitivity, specificity and efficiency. The MIP-LAMP approach was then successfully used for the rapid, specific and sensitive detection of *L. monocytogenes* targeting the *L. monocytogenes*—specific *hlyA* gene, which encodes a cholesterol-dependent cytolysin responsible for clinical symptoms and is highly conserved in *L. monocytognes* [22]. Furthermore, the novel MIP-LAMP assay was compared with normal LAMP (nLAMP) technique by testing the LoD levels and the practical application was successfully evaluated by detecting the target pathogen in pork samples.

## 2. Results

### 2.1. The Principle of the MIP-LAMP for Detecting Target DNA

**Figure 1:**
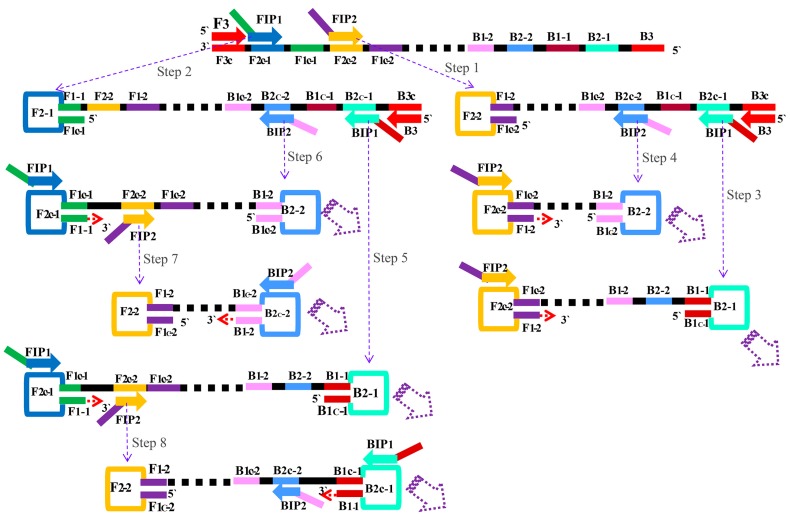
The principle of MIP-LAMP amplification. The schematic showing the mechanism of the novel MIP-LAMP assay.

The basic principle of the MIP-LAMP assay is illustrated in Figure 1. The MIP-LAMP reaction requires a DNA polymerase with strand displacement activity and a set of six to ten specially designed primers, which consists of two outer primers (F3 and B3), four inner primers (FIP1, FIP2, BIP1 and BIP2) or four loop primers (LF1, LF2, LB1 and LB2). The four inner primers contain sequences of the sense and antisense strands of the target DNA. Inner primers FIP2 and BIP2 initiate MIP-LAMP amplification (Step 1) and will be displaced when the DNA polymerase extends upstream FIP1 and BIP1 primers (Step 2). Two outer primers displace the latter amplification strands and release single-stranded DNAs with FIP1 and BIP1. As a result, several distinct types of single-stranded DNAs form hairpin structures to initiate the loops for cyclic amplification (Step 3–8). For next elongation, the amplification process is similar to nLAMP, each strand is displaced, and in every cycle, the new stem-loops are synthesized. The final products are the stem-loop DNA fragments with several inverted repeats of target DNA and cauliflower-like structures with multiple loops, which are produced in the process of hybridization between alternately inverted repeats present in the same strand. Furthermore, four loop primers are added into the reaction mixtures to accelerate the MIP-LAMP reaction, which do so by identical nLAMP mechanisms.

### 2.2. Primers Design of MIP-LAMP

**Figure 2:**
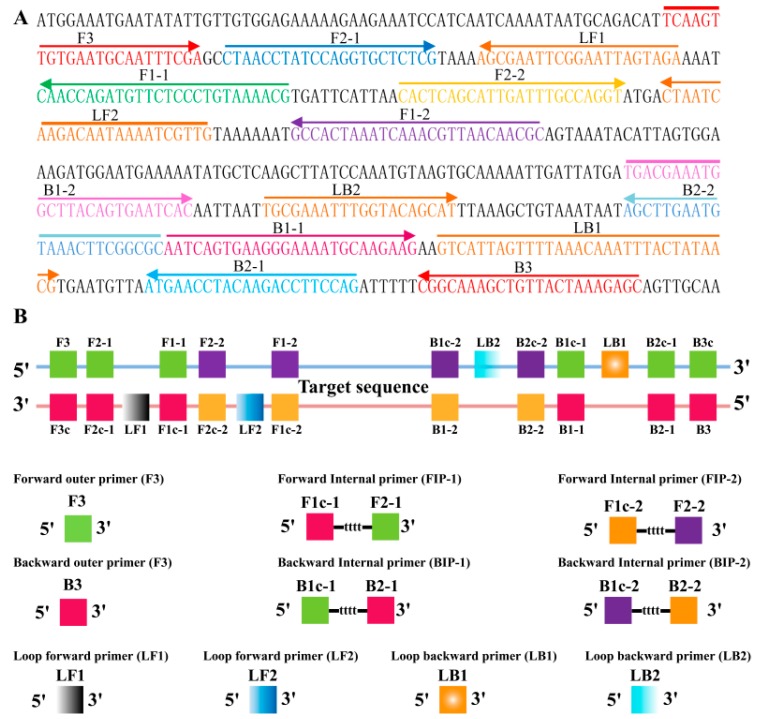
Primers design of MIP-LAMP. (**A**), location and orientation of *L. monocytogenes* specific MIP-LAMP primers within the nucleotide sequence of the listeriolysin O (*hlyA*) gene (GenBank accession number M24199). The nucleotide sequences of the primer sites are underlined, left and right arrows indicate complementary and sense sequences that are used; (**B**), diagram exhibiting the positions at which the primers attach to amplify the target sequence.

For the *L. monocytogenes*—specific *hlyA* gene, a set of 10 primers, which targeted 14 distinct regions, was designed for MIP-LAMP by using PrimerExplorer V4 (Eiken Chemical, Co., Ltd., Tokyo, Japan) and the primer software Primer Premier 5.0. Forward inner primers FIP1/FIP2 consist of the complementary sequence of F1c-1/F1c-2, a T-T-T-T linker, and F2-1/F2-2; Backward inner primers BIP1/BIP2 consist of the complementary sequence of B1c-1/B1c-2, a T-T-T-T linker, and B2-1/B2-2. The outer primers F3 and B3 are located outside of the F2-1 and B2-1 regions, while loop primers LF1, LF2, LB1 and LB2 are located between F2-1 and F1-1, F2-2 and F1-2, B2-1 and B1-1, or B2-2 and B1-2, respectively (Table 1; Figure 2).

**Table 1:** Primers used for MIP-LAMP amplification.

Primers	Sequence (5′-3′)	Length
F3	TCAAGTTGTGAATGCAATTTCGA	23 nt
B3	GCTCTTTAGTAACAGCTTTGCCG	23 nt
FIP1(F1c-1 + F2-1)	CGTTTTACAGGGAGAACATCTGGTTG*tttt*CTAACCTATCCAGGTGCTCTCG	52 mer
FIP2(F1c-2 + F2-2)	GCGTTGTTAACGTTTGATTTAGTGGC*tttt*CACTCAGCATTGATTTGCCAGGT	53 mer
BIP2(B1c-1 + B2-1)	TGACGAAATGGCTTACAGTGAATCAC*tttt*GCGCCGAAGTTTACATTCAAGCT	53 mer
BIP1(B1c-2 + B2-2)	AATCAGTGAAGGGAAAATGCAAGAAG*tttt*CTGGAAGGTCTTGTAGGTTCAT	52 mer
LF1	TCTACTAATTCCGAATTCGCT	21 nt
LF2	CAACGATTTTATTGTCTTGATTAG	24 nt
LB2	TGCGAAATTTGGTACAGCAT	20 nt
LB1	GTCATTAGTTTTAAACAAATTTACTATAACG	31 nt
P1	CTAACCTATCCAGGTGCTCTCG	22 nt
P2	CACTCAGCATTGATTTGCCAGGT	23 nt
B2	GCGCCGAAGTTTACATTCAAGCT	23 nt
B1	CTGGAAGGTCTTGTAGGTTCAT	22 nt

### 2.3. The Availability of MIP-LAMP Primers

**Figure 3:**
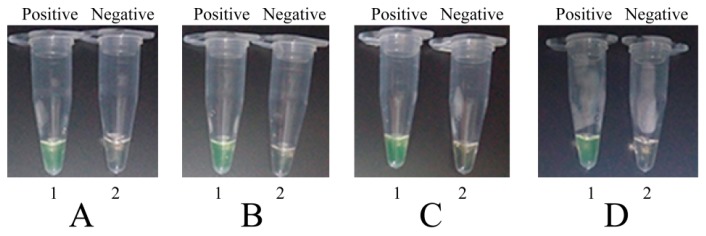
Amplification products of 4 nLAMP assays were visually detected by FD. (**A**–**D**): nLAMP assay using primers set G1, G2, G3 and G4, respectively. Tube 1, positive amplification, tube 2, negative amplification.

**Figure 4:**
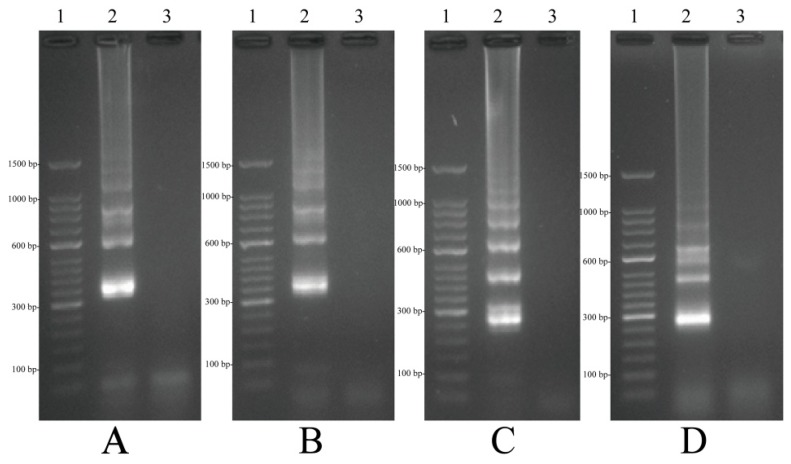
Agarose gel electrophoresis of 4 nLAMP products. (**A**–**D**): nLAMP assay using primers set G1, G2, G3 and G4, respectively. Lane 1, DL 50-bp DNA marker; lane 2, positive nLAMP products; lane 3 negative control.

To determine whether a set of 10 primers was available for MIP-LAMP amplification, four separate nLAMP reactions were carried out with primers set G1, G2, G3 and G4 (Experimental Section 4.3), which were incubated at 64 °C for 1 h. Based on visual detection with FD, positive or negative results were easily determined. All positive amplification appeared green, while negative controls remained light gray (Figure 3). The typical ladder-liker pattern bands on 2.5% gel electrophoresis were generated in positive amplification but not in the negative controls (Figure 4). These results indicated that the set of 10 primers were available for MIP-LAMP reaction.

### 2.4. Confirmation and Detection of MIP-LAMP Products

**Figure 5:**
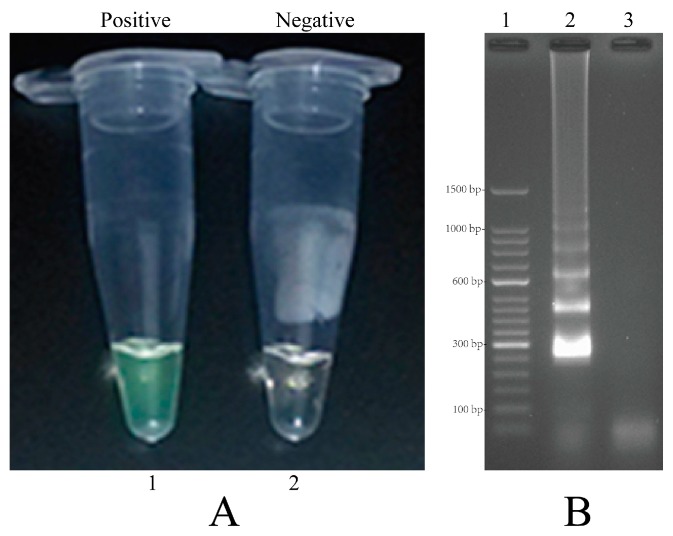
Amplification products of MIP-LAMP assays were visually detected both by FD and agarose gel electrophoresis. (**A**): color change of MIP-LAMP tubes; tube 1, positive amplification; tube 2, negative amplification; (**B**): agarose gel electrophoresis of MIP-LAMP products; lane 1, DL 50-bp DNA marker; lane 2, positive nLAMP products; lane 3 negative control.

In order to demonstrate the mechanism of MIP-LAMP, amplification reactions were carried out in the presence of or absence of genomic DNA templates. The positive amplification tube, which contained the DNA templates, was indicated by a colour change from light gray to green, while the negative control remained light gray (Figure 5A). The positive reaction by electrophoresis showed a ladder-liker pattern after 2.5% agarose gel electrophoresis analysis but not in the negative controls (Figure 5B). The result showed that our MIP-LAMP approach was feasible for DNA amplification.

### 2.5. Validation of the Reliability of MIP-LAMP by Sequencing

**Figure 6:**
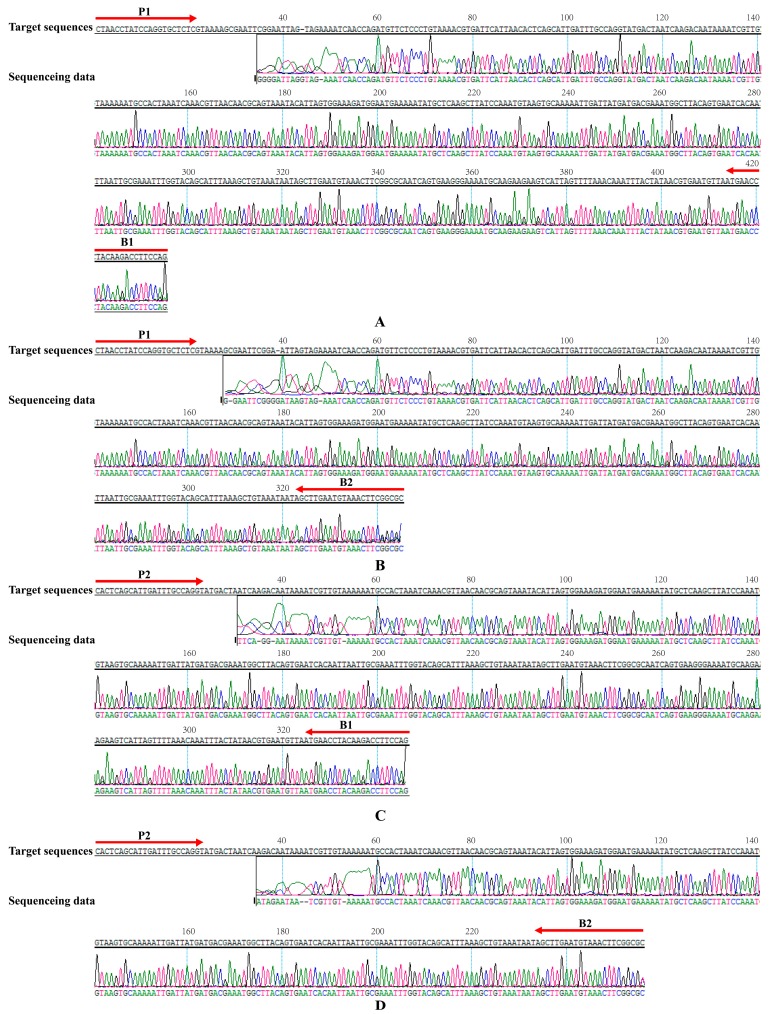
Sequencing analysis of MIP-LAMP amplified *L. monocytogenes* *hlyA* gene. The target sequences were presented at the top and the sequences of the primers sites (P1, P2, B1 and B2) were underlined. (**A**–**D**): the sequencing data were obtained from primers P1 and B1, P1 and B2, P2 and B1, P2 and B2, respectively. Left and right arrows indicated complementary and sense sequences that were used. The sequencing data were shown in the bottom.

In order to further confirm the reliability and specificity of MIP-LAMP assay, a MIP-LAMP-PCR-sequencing strategy was established to analyze the MIP-LAMP products. The sequences of PCR amplicons, which amplified from MIP-LAMP products, were 97% match with the expected target sequences (Figure 6). The sequencing data indicated that the correct amplification of MIP-LAMP was further verified.

### 2.6. The Optimal Temperature of MIP-LAMP Assay

**Figure 7:**
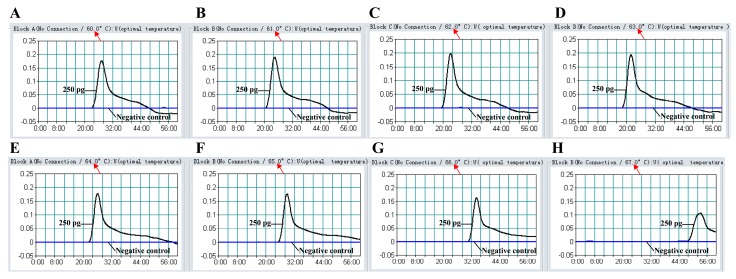
The optimal temperature for the MIP-LAMP assay. The MIP-LAMP amplifications reactions were analyzed by real-time measurement of turbidity and the corresponding curves of concentrations of DNA were marked in the Figure. The threshold value was 0.1 and the turbidity of >0.1 was considered to be positive. Eight kinetic graphs (**A**–**H**) were obtained at different temperature (60–67 °C) with *L. monocytogenes* DNA at the level of 2.5 pg per reaction, and the graphs from B to F showed robust amplification.

**Figure 8:**
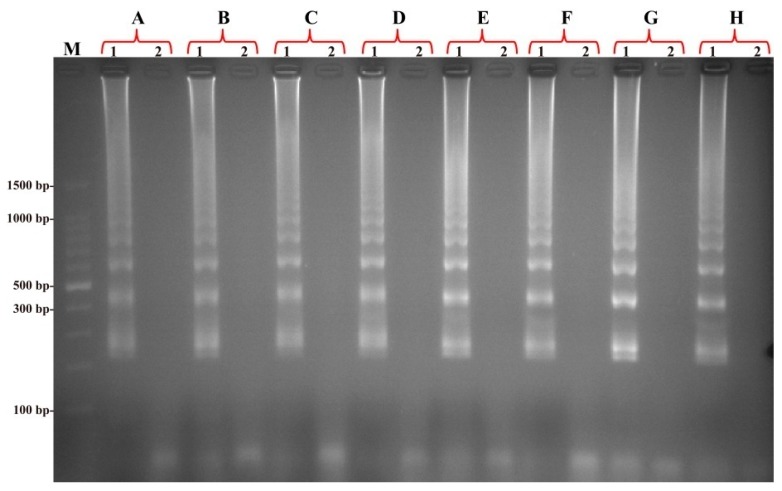
Products of MIP-LAMP monitored using 2.5% agarose gel electrophoresis. The products (**A**–**H**) of MIP-LAMP from different reaction temperature (60–67 °C) were monitored by 2.5% agarose gel electrophoresis after staining with ethidium bromide. Lane M, DL 100-bp DNA marker; lane 1, positive MCDA products; lane 2, negative control (no DNA).

To determine the optimal temperature of MIP-LAMP reaction, the reference strain EGD-e was selected as positive control at the level of 2.5 pg genomic DNA per reaction and the MIP-LAMP reactions were carried out at different temperatures (60–67 °C) with the appropriate primers. The results were monitored by real-time measurement of turbidity and a typical kinetics graph was shown in Figure 7. Moreover, the positive amplifications were also observed as a ladder-like banding pattern on agarose gel electrophoresis (Figure 8). The amplification temperatures of 61–66 °C were recommended as the standard temperatures for the MIP-LAMP methodology and the temperature of 63 °C was used for the rest of MIP-LAMP reaction conducted in this study.

### 2.7. The Specificity of the MIP-LAMP Assay for Detection of L. monocytogenes

**Figure 9:**
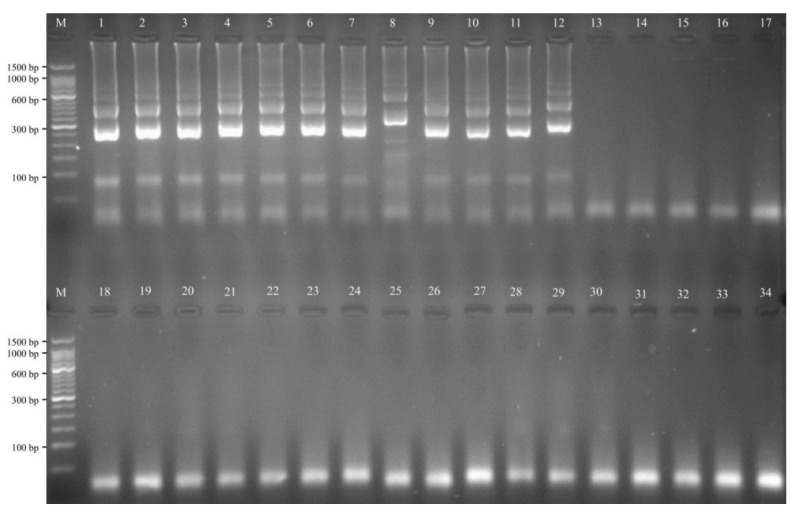
Specificity of MIP-LAMP detection for different strains. Lane M, DL 50-bp DNA marker; lane 1–12, *L. monocytogenes* strains of serovar 1/2a (EGD-e), 3a (ICDCLM023), 1/2c (ICDCLM010), 3c (ICDCLM446), 1/2b (ICDCLM007), 3b (ICDCLM078), 7 (NCTC10890), 4a (ATCC19114), 4c (ATCC19116), 4b (ICDC419), 4d (ATCC19117) and 4e (ATCC19118); lane 13–17, other *Listeria* reference strains of *L. ivanovii* (ATCCBAA-678), *L. innocua* (ATCCBAA-680), *L. grayi* (ATCC25402), *L. seeligeri* (ATCC35967), *L. welshimeri* (ATCC35897); lane 18–34, non-*Listeria* strains of *Bacillus cereus*, *Enteropathogenic E. coli*, *Enterotoxigenic E. coli*, *Enteroaggregative E. coli*, *Enteroinvasive E. coli*, *Enterohemorrhagic E. coli*, *Plesiomonas* *shigelloides*, *Shigella* *flexneri*, *Shigella* *sonnei*, *Enterobacter cloacae*, *Enterococcus faecalis*, *Yersinia enterocolitica*, *Aeromonas* *hydrophila*, *Citrobacter* *freumdii*, *Proteus vulgaris*, *Vibrio fluvialis*, *Salmonella enterica*.

Detection specificity of the *L. monocytogenes* MIP-LAMP assay was evaluated by using various bacterial strains. Thirty nine *L. monocytogenes* DNA templates were correctly identified, whereas no amplification of the 34 non-*L. monocytogenes* templates was observed (Figure 9). These results demonstrated that the MIP-LAMP method employing a set of 10 primers had high selectivity for screening *L. monocytogenes*.

### 2.8. The Analytical Sensitivity of MIP-LAMP Assays

**Figure 10:**
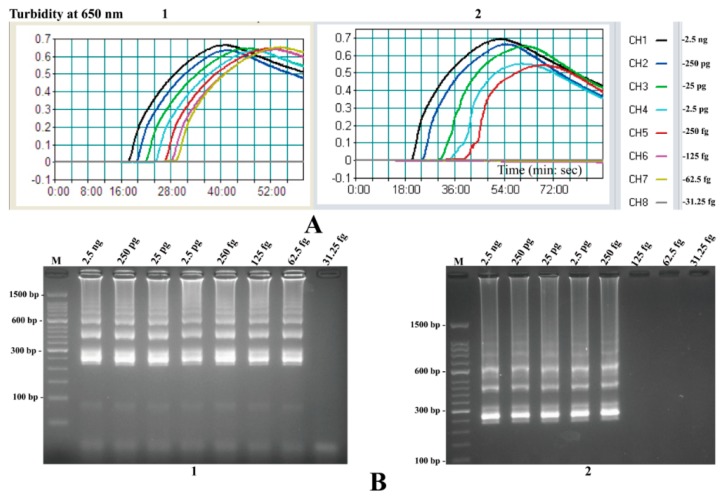
Sensitivity of the MIP-LAMP and nLAMP assays using serially diluted genomic DNA with *L. monocytogenes* strain EGD-e as template. **A**, Sensitivity of MIP-LAMP (**A1**) and nLAMP (**A2**) for *L. monocytogenes* detection was analyzed by real-time measurement of turbidity. The LoD for MIP-LAMP and nLAMP assays were 62.5 fg and 250 fg genomic DNA per reaction, respectively; (**B**), Sensitivity of MIP-LAMP (**B1**) and nLAMP (**B2**) approaches for *L. monocytogenes* detection were seen by gel electrophoresis, respectively. Lane M, DL 50-bp DNA marker. The positive results were observed as a ladder-like pattern on 2.5% agarose gel electrophoresis analysis.

To test the LoD of the MIP-LAMP assays, serial dilutions (from 2.5 ng to 31.25 fg) of total genomic DNA extracted from pure-cultured *L. monocytogenes* were subjected to MIP-LAMP reactions in triplicate. The MIP-LAMP amplifications were monitored by real-time turbidity detection (Realtime Turbidimeter LA-320C, Eiken), and the decreasing concentrations of genomic DNA were presented from left to right (Figure 10). The minimum detection concentration required for the MIP-LAMP assay was 62.5 fg genomic DNA per reaction. Agarose gel electrophoresis of the MIP-LAMP products, which was used as alternative detection method, displayed the typical ladder-like patterns in positive tubes (Figure 10). Moreover, the *hlyA*-MIP-LAMP amplifications required only 35-min incubation periods at LoD levels of DNA templates.

### 2.9. Sensitivity Comparison of nLAMP and MIP-LAMP in Test of Genomic DNA of L. monocytogenes

The LoD of nLAMP assays were found to be 250 fg genomic DNA per reaction for *L. monocytogenes*, while the novel MIP-LAMP assay was able to amplify DNA at lower levels (125 and 62.5 fg) of genomic DNA per reaction (Figure 10). The results demonstrated that *hlyA*-MIP-LAMP assay was 4-fold more sensitive than *hlyA*-LAMP approach for detecting *L. monocytogenes* genomic DNA. Moreover, the sensitivity of the novel MIP-LAMP methodology on *L. monocytogenes* was evaluated by detecting CFUs, and the LoD of MIP-LAMP technique for *hlyA* gene with pure cultures was 2.4 CFU per reaction (The MIP-LAMP reaction was found to be positive for sample containing 2.4 × 10^3^ CFU per milliliter, with 1 μL was included in the MIP-LAMP amplification system) (data not shown). However, the sensitivity of nLAMP reactions set for detection of *L. monocytogenes* in pure cultures were 24 CFU per reaction, thus these results indicated that the *hlyA*-MIP-LAMP assay was 10-fold more sensitive than *hlyA*-nLAMP methods for detecting *L. monocytogenes* CFUs.

### 2.10. Evaluation of the MIP-LAMP Assay in Artificially Contaminated Milk

To examine the applicability of the novel MIP-LAMP technology as a surveillance tool for *L. monocytogenes*, comparative analysis of sensitivity of *L. monocytogenes* detection by MIP-LAMP and nLAMP was performed using a dilution series of artificially contaminated milk. The LoD of the MIP-LAMP assay was found to be 24 cells per reaction which corresponds to 2.4 × 10^4^ CFU per milliliter, while the nLAMP method had a detection limit of 2.4 × 10^5^ CFU/mL for *hlyA* gene in artificial contamination of milk samples (data not shown).

### 2.11. Evaluation of the MIP-LAMP Assay Using Pork Samples

To evaluate the feasibility of the novel MIP-LAMP assay to detect *L. monocytognes* in food samples, 48 pork samples were analyzed using the novel MIP-LAMP approach, compared to the nLAMP and culture-based methods. The results are summarized in Table 2. Of a total of 48 pork samples, 13 and 13 were tested to be positive by MIP-LAMP and nLAMP in 48-h FB broth, while 13 and 10 were detected to be positive by MIP-LAMP and nLAMP in 24-h FB broth, respectively. The detection accuracy of the MIP-LAMP assay was 100% when compared with the culture-biotechnical method, indicating the high specificity and sensitivity of the novel MIP-LAMP.

**Table 2:** Comparison of culture-biotechnical, nLAMP and MIP-LAMP for the detection of *L. monocytogenes* in pork samples.

Samples	Cultures	nLAMP		MIP-LAMP	
		24 h in FB	48 h in FB	24 h in FB	48 h in FB
1	−	−	−	−	−
2	−	−	−	−	−
3	−	−	−	−	−
4	−	−	−	−	−
5	−	−	−	−	−
6	+	+	+	+	+
7	−	−	−	−	−
8	−	−	−	−	−
9	−	−	−	−	−
10	−	−	−	−	−
11	+	+	+	+	+
12	−	−	−	−	−
13	−	−	−	−	−
14	+	+	+	+	+
15	+	+	+	+	+
16	−	−	−	−	−
17	−	−	−	−	−
18	+	+	+	+	+
19	+	+	+	+	+
20	+	−	+	+	+
21	−	−	−	−	−
22	−	−	−	−	−
23	−	−	−	−	−
24	−	−	−	−	−
25	−	−	−	−	−
26	−	−	−	−	−
27	−	−	−	−	−
28	−	−	−	−	−
29	+	−	+	+	+
30	−	−	−	−	−
31	−	−	−	−	−
32	−	−	−	−	−
33	−	−	−	−	−
34	−	−	−	−	−
35	−	−	−	−	−
36	−	−	−	−	−
37	−	−	−	−	−
38	−	−	−	−	−
39	−	−	−	−	−
40	−	−	−	−	−
41	+	+	+	+	+
42	+	+	+	+	+
43	−	−	−	−	−
44	+	−	+	+	+
45	−	−	−	−	−
46	+	+	+	+	+
47	−	−	−	−	−
48	+	+	+	+	+
Total	13	10	13	13	13

## 3. Discussion

The most conventional technologies for screening and detecting foodborne pathogens are to use bacteriological media to selectively grow and enumerate bacteria. Although inexpensive and effective, these approaches are culture-dependent and the enrichment procedures take at least 24 h [23]. Beside, preparation of microbiological media and bacteriological plates, as well as colony counting and biochemical identifications of the isolated colonies are laborious and time-consuming processes. Thus, one of the best methods to detect target pathogens is to employ nucleic acid-based techniques to guarantee high selectivity. Among the most promising and attractive technologies, the PCR and PCR-based assays are the most widespread techniques in screening target pathogens due to its rapidness, sensitivity and specificity [24,25]. However, expensive apparatus and sophisticated technical skill required to conduct these techniques make them inaccessible in resource-poor settings, what’s more, problems with the *in situ* applications have hampered its routine use in many laboratories [26,27]. Therefore, it is urgently necessary to devise a simple, rapid and cost-effective alternative solution to microbiological examination. 

Loop-mediated isothermal amplification (LAMP), a powerful innovative gene amplification technique, has been described as an easy and rapid diagnostic tool for the detection of microbes and viruses, but still has room for improvement in a number of areas [28]. The approach may not detect extremely low levels of microorganisms that are non-culturable cells present in clinical specimens and food samples, such as sublethally injured bacteria [21,29,30]. Thus, we presented the novel MIP-LAMP methodology, as an isothermal amplification strategy, which did not require thermal denaturation of templates, and obviating the use of the temperature-regulating apparatus. The FD colorimetric indicator, a visual detection technique for the results of the novel MIP-LAMP amplifications, shows visual discrimination of the positive results within 35-min, eliminating the use of costly specialized equipment and further procedures.

As a technological improvement, we modified the conventional LAMP technique by employing two additional inner primers and loop primers that significantly enhance the sensitivity and shorten the amplification time. The MIP-LAMP assay developed in this study was capable of detecting 62.5 fg *L. monocytogenes* DNA per reaction and 2.4 CFU per reaction in pure culture within 45-min incubation period, which was more sensitive than the nLAMP assay (Figure 10, A1 and B1). Moreover, the newly developed MIP-LAMP technology detected as little as 24 CFU per reaction in artificially contaminated milk samples and was also more sensitive than the conventional LAMP methodology. The analytical sensitivity of the novel MIP-LAMP technique was 16-fold more sensitive than the *hlyA*-LAMP assay for detection of *L. monocytogenes*, which was reported by Wang *et al.* [31]. Furthermore, six core primers (two outer primers and four inner primers) with ten binding sites hybridize correctly to their target sequences which ensure the higher specificity of MIP-LAMP, and two pairs of loop primers are added to the reaction to further enhance the amplification of MIP-LAMP. Similar to LAMP assay, the MIP-LAMP products can be analyzed by gel electrophoresis, FD reagents and real-time turbidimetry of reaction tubes [32,33]. With these advantages, the novel MIP-LAMP has the potential to be widely used as a diagnostic tool. Therefore, these attractive properties can motivate researchers to explore the use of the novel MIP-LAMP for genetic analysis in diverse fields.

Listeriosis cases are associated with the digestion of unhygienic food products, post-processing contaminated food and ready-to-eat food [34]. In order to quickly identify the source of foodborne outbreaks or for a faster commercial batch release, the diagnostic tools for rapid detection and differentiation of the target pathogen in food are required. In this present study, the novel MIP-LAMP technique was applied to amplify the species-specific *hlyA* gene for detection and identification of foodborne *L. monocytognes* and the diagnostic sensitivity and specificity of the method were successfully assessed by using a panel of bacterial DNA templates and pork samples collected from the local markets. The results demonstrated that the novel MIP-LAMP assay for screening *L. monocytognes* was sensitive (62.5 fg genomic DNA per reaction in pure culture), specific (no false-positive amplification for the strains tested in the study) and rapid (within 45-min incubation periods) (Figure 10). In order to achieve the requirement of samples detection, the practical application of MIP-LAMP detection to *L. monocytogenes* in pork samples was evaluated. Forty eight pork samples were collected from the local markets and examined by the novel MIP-LAMP, nLAMP and culture-technical detection for the presence of *L. monocytongenes*. Thirteen of the 48 pork samples were positive for *L. monocytogenes* using the novel MIP-LAMP assay with a 24 h-enrichment in FB, and the results were consistent with culture-biotechnical methods. Comparing to the culture-technical assay, the novel MIP-LAMP technique saved time and was less expensive. Three pork samples were confirmed to be positive by MIP-LAMP and culture-biotechnical detection, but negative by using nLAMP assay with a 24 h-enrichment in FB. Therefore, the *L. monocytognes* MIP-LAMP-based assay, which provides the analytical advantages on rapid amplification, high specificity and sensitivity, can be used as a potential screening tool for *L. monocytogenens* in routine food safety monitoring.

## 4. Experimental Section

### 4.1. Primers for the MIP-LAMP Amplification

A set of 10 primers, including six core primers (F3, B3, FIP1, FIP2, BIP2 and BIP1) and four loop primers (LF1, LF2, LB2 and LB1) were designed on the basis of the mechanism of MIP-LAMP for detection of *L. monocytogenes* (Figure 1). The locations and sequences of the MIP-LAMP primers are shown in Figure 2 and Table 1.

### 4.2. Bacterial Strains and Genomic DNA Extraction

A total of 73 bacterial stains, including 39 *L. monocytogenes* and 34 non-*L. monocytogenes* strains, were used in the inclusivity and exclusivity tests, respectively (Table 3). All *Listeria* and other non-*Listeria* strains were grown on brain heart infusion plates overnight at 37 °C. Strain EGD-e was selected as a positive control for the assay of optimization and sensitivity testing with pure culture.

Bacterial genomic DNA was extracted from all cultured strains using DNA extraction kits (QIAamp DNA minikits; Qiagen, Hilden, Germany) according to the manufacturer’s instructions. DNA samples were stored at −20 °C.

**Table 3:** Bacterial strains used in the study.

Bacteria	Serovar ^a^	Strain No. (Source of Strain) ^b^	No. of Strains
*Listeria monocytogenes*	1/2a	EGD-e	1
		Isolated strains (ICDC)	4
	3a	Isolated strains (ICDC)	5
	1/2b	Isolated strains (ICDC)	5
	3b	Isolated strains (ICDC)	1
	1/2c	Isolated strains (ICDC)	5
	3c	Isolated strains (ICDC)	1
	7	NCTC10890	1
	4a	ATCC19114	1
		Isolated strains (ICDC)	2
	4c	ATCC19116	1
		Isolated strains (ICDC)	2
	4b	Isolated strains (ICDC)	5
	4d	ATCC19117	1
		Isolated strains (ICDC)	2
	4e	ATCC19118	1
		Isolated strains (ICDC)	1
*Listeria ivanovii*	U	ATCCBAA-678	1
		Isolated strains (ICDC)	2
*Listeria innocua*	U	ATCCBAA-680	1
		Isolated strains (ICDC)	2
*Listeria grayi*	U	ATCC25402	1
		Isolated strains (ICDC)	2
*Listeria seeligeri*	U	ATCC35967	1
		Isolated strains (ICDC)	1
*Listeria welshimeri*	U	ATCC35897	1
		Isolated strains (ICDC)	2
*Bacillus cereus*	U	Isolated strains (ICDC)	1
*Enteropathogenic E. coli*	U	Isolated strains (ICDC)	1
*Enterotoxigenic E. coli*	U	Isolated strains (ICDC)	1
*Enteroaggregative E. coli*	U	Isolated strains (ICDC)	1
*Enteroinvasive E. coli*	U	Isolated strains (ICDC)	1
*Enterohemorrhagic E. coli*	U	EDL933	1
*Plesiomonas* *shigelloides*	U	Isolated strains (ICDC)	1
*Shigella* *flexneri*	U	Isolated strains (ICDC)	1
*Shigella* *sonnei*	U	Isolated strains (ICDC)	1
*Enterococcus faecalis*	U	ATCC35667	1
*Enterococcus faecium*	U	Isolated strains (ICDC)	1
*Yersinia enterocolitica*	U	Isolated strains (ICDC)	1
*Aeromona* *shydrophila*	U	ATCC7966	1
*Citrobacter* *freumdii*	U	Isolated strains (ICDC)	1
*Proteus vulgaris*	U	Isolated strains (ICDC)	1
*Vibrio fluvialis*	U	Isolated strains (ICDC)	1
*Streptococcus bovis*	U	Isolated strains (ICDC)	1
*Vibrio parahaemolyticus*	U	Isolated strains (ICDC)	1
*Klebsiella* *pneumoniae*	U	Isolated strains (ICDC)	1
*Salmonella enterica*	U	Isolated strains (ICDC)	1

^a^ U, unidentified serotype. ^b^ ATCC, American Type Culture Collection; NCTC, National Collection of Type Cultures; ICDC, National Institute for Communicable Disease Control and Prevention, China CDC.

### 4.3. The MIP-LAMP Assay

The MIP-LAMP detection system consists of four sets of nLAMP primers, which are named as G1 (F3, B3, FIP1, LF1, BIP1 and LB1), G2 (F3, B3, FIP1, LF1, BIP2 and LB2), G3 (F3, B3, FIP2, LF2, BIP1 and LB1) and G4 (F3, B3, FIP2, LF2, BIP2 and LB2). In order to demonstrate the availability of MIP-LAMP primers, the nLAMP reactions were performed by using G1, G2, G3 and G4 primers set.

The four nLAMP methods were performed with the Loopamp DNA amplification Kit (Eiken Chemical Co. Ltd.) in a final volume of 25 μL containing 1.6 μM each of primers FIP1 (or FIP2) and BIP1 (or BIP2), 0.8 μM each of primers LF1 (or LF2) and LB1 (or LB2), 0.2 μM each of primers F3 and B3, 12.5 μL 2× reaction mix, 1 μL of *Bst* DNA polymerase (8 U), 1 μL Loopamp^®^ Fluorescent Detection Reagent (FD) and 1 μL DNA template.

The MIP-LAMP amplification was also carried out in a total 25-μL reaction mixture containing 0.4 μM each of primers F3 and B3, 1.6 μM each of primers FIP1 and BIP1, 0.6 μM each of primers LF1 and LB1, 2.0 μM each of primers FIP2 and BIP2, 0.8 μM each of primers LF2 and LB2, 12.5 μL 2× reaction mix, 1.25 μL of *Bst* DNA polymerase (10 U), 1 μL FD and 1 μL DNA template.

The 25 μL reaction mixtures of nLAMP and MIP-LAMP were incubated at 64 °C for 60 min and then heated at 95 °C for 5 min to stop the reaction. A sample was used as a negative control, in which no template was added. A total of 3 methods were used to confirm nLAMP and MIP-LAMP DNA amplification, including direct visual inspection of the amplification products with FD by naked eye, electrophoresis in 2.5% agarose gels with ethidium bromide staining, and real-time measurement of turbidity using Loopamp Real-time Turbidimeter LA-320C (Eiken Chemical Co., Ltd.). These techniques were used to analyze that nLAMP and MIP-LAMP tests amplified the correct targets. Analysis of each sample was tested at least three times.

In order to evaluate the optimal reaction temperature, the MIP-LAMP amplification was carried out at a constant temperature ranging from 60 °C to 67 °C for 60 min in 1 °C increments in separate reactions and then incubated at 85 °C for 10 min to stop the reaction. Mixture without DNA template was used as a negative control.

### 4.4. Validation by Sequencing

To further evaluate the reliability and specificity of the MIP-LAMP approach, a MIP-LAMP-PCR-sequencing was devised to analyze the MIP-LAMP products. The MIP-LAMP products corresponding to the level of 62.5 fg template DNA were confirmed by agarose gel electrophoresis, and the ladders between 100 bps and 1500 bps were extracted and purified by using QIAquick Gel Extraction Kit (Qiagen) to obtain the template DNA for PCR. Four sets of primers (P1, B1; P1, B2; P2, B1 and P2, B2) were used (Table 1), and PCR reactions were carried out in a final volume of 20 μL containing 10 mMTris-HCl (pH 8.3), 50 mMKCl, 1.5 mM MgCl_2_, 0.001% gelatin, 0.2 μM each of P1, P2, B1 and B2 primers, 0.2 mM each of dNTPs, 0.5 μL DNA template, and 0.5 units of Taq DNA polymerase (ExTaq; Takara, Dalian, China). The amplification program consisted of the initial denaturation of 5 min at 95 °C, 35 cycles of 30 s at 95 °C, 30 s at 58 °C and 1 min at 72 °C, plus a final 5 min extension at 72 °C. The PCR products of each reaction were also validated by electrophoresis, and then extracted and purified by using QIAquick Gel Extraction Kit (Qiagen) again to obtain the template for sequencing. Sequencing was performed using the same primers as for PCR (Tsingke, Beijing, China) and the sequence data were compared with the target gene sequences in the GenBank database.

### 4.5. The MIP-LAMP Specificity Test

To evaluate the specificity of the MIP-LAMP assays, the MIP-LAMP reactions were carried out under the conditions described above with DNA templates from 39 *L. monocytogenes* and 34 non-*L. monocytogenes* strains (Table 2). Analysis of each sample were examined twice independently.

### 4.6. The MIP-LAMP Sensitivity Test

In order to determine the LoD of MIP-LAMP assay, the sensitivity was examined using serial dilutions of genomic DNA templates (2.5 ng, 250 pg, 25 pg, 2.5 pg, 250 fg, 125 fg, 62.5 fg and 31.25 fg, respectively). To test the minimal detectable colony forming units (CFUs), the cultures with EGD-e strain were serially diluted (10^−1^ to 10^−9^), and aliquots of 100 μL of each dilution were used to extract DNA as described above. Moreover, the aliquots of 100 μL appropriate dilution (10^−6^) was plated in triplicate on brain heart infusion (BHI) and the CFUs were counted after 24 h at 37 °C. The analytical sensitivity comparison of nLAMP and MIP-LAMP was also carried out with the identical dilutions of templates at the optimal conditions in triplicate. The *hlyA*-LAMP assay has been developed by Tang *et al.*, which were employed to verify the LoD of nLAMP approaches [35].

### 4.7. MIP-LAMP Application in Artificially Contaminated Milk

The milk was purchased from a grocery store in Beijing and confirmed as being *L. monocytogenes*—negative by traditional culture assay and PCR [36]. It was artificially contaminated with *L. monocytogenes* strain EGD-e at five concentrations (2.4 × 10^5^, 2.4 × 10^4^, 2.4 × 10^3^, 2.4 × 10^2^ and 2.4 × 10^1^ CFUs per milliliter), individually, and aliquots (100 μL) of the milk were used for DNA extraction. This experiment was performed in triplicate independently, and the supernatants (1 μL) were used for MIP-LAMP and nLAMP detection.

### 4.8. Practical Application of MIP-LAMP Detection to L. monocytogenes in Food Samples

To estimate the feasibility of the MIP-LAMP assay to detect *L. monocytogenes* in food samples, we tested 48 pork samples collected from local market, and compared the results with a culture-biotechnical method, MIP-LAMP and nLAMP detection for the same samples. According to the ISO 11290-1 standard method, culture-biotechnical method of food samples was carried out. In brief, an amount of 25 g from each pork sample was added to a bag containing 225 mL *Listeria* enrichment broth (Half Fraser’s broth, Oxoid, Hampshire, UK) and homogenized, followed by incubating broth for 24 h at 30 °C. Then, 0.1 mL of the enrichment broth was transferred to 10 mL of Fraser’s broth (FB) in a culture tube, which was incubated for 24 and 48 h at 37 °C with shaking (250 rpm). Aliquots (1 mL) of the enriched culture were subjected to DNA extraction and used as templates for nLAMP and MIP-LAMP detections. A portion (0.05 mL) of positive FB enriched cultures after 48 h incubation was plated on PALCAM agar plates (Oxoid), which were incubated for 48 h at 37 °C. Five typical *Listeria* colonies with an opaque halo were selected from each of selective plates and the further confirmation of *L. monocytogenes* strains were identified by characteristic colony morphology, gram stain, and various tests (motility, aesculin hydrolysis, catalase, indole, urease, CAMP and oxidase tests).

## 5. Conclusions

A novel MIP-LAMP technology was established for amplification of the target sequences, which was successfully applied to detect *hlyA* gene in *L. monocytogenes*. The MIP-LAMP methodology developed here is easy, rapid and robust, which has advantages over nLAMP assay, namely more rapid amplification, higher specificity and sensitivity. In the analysis of food samples, the novel MIP-LAMP assay has the potential to be a rapid, sensitive, and cost-effective for detection of *L. monocytogenes* in food. Eventually, it is anticipated that the novel MIP-LAMP assay has enormous potential for detection of a variety of bio-related markers.
